# Shp2 Inhibits Proliferation of Esophageal Squamous Cell Cancer via Dephosphorylation of Stat3

**DOI:** 10.3390/ijms18010134

**Published:** 2017-01-12

**Authors:** Chen Qi, Tao Han, Hua Tang, Kenan Huang, Jie Min, Jing Li, Xinyu Ding, Zhifei Xu

**Affiliations:** 1 Department of Thoracic Surgery, Shanghai Changzheng Hospital, Second Military Medical University, Shanghai 200003, China; cqi@smmu.edu.cn (C.Q.); htang@smmu.edu.cn (H.T.); knhuang@smmu.edu.cn (K.H.); jmin@smmu.edu.cn (J.M.); 2 Department of Oncology, Cancer Center, People’s Liberation Army General Hospital of Shenyang Military Region, Shenyang 110016, China; than1984@sina.com (T.H.); crzs281@tom.com (J.L.)

**Keywords:** esophageal squamous cell cancer, proliferation, Shp2

## Abstract

Shp2 (Src-homology 2 domain-containing phosphatase 2) was originally reported as an oncogene in kinds of solid tumors and hematologic malignancies. However, recent studies indicated that Shp2 may act as tumor suppressors in several tumor types. We investigated the function of Shp2 in esophageal squamous cell cancer (ESCC). The expression level of Shp2 was analyzed in tumor tissues in comparison with adjacent normal tissues of ESCC patients by immunohistochemistry and Western blot. Shp2 was knocked down by Short hairpin RNA to evaluate its function in ESCC cell lines. The relationship between Shp2 and p-Stat3 (signal transducer and activator of transcription 3) in human ESCC tissues was statistically examined. A significant low expression of Shp2 was found in ESCC tissues. Low expression of Shp2 was related to poorer overall survival in patients from The Cancer Genome Atlas (TCGA) dataset. Knockdown of Shp2 increased the growth of ESCC cell lines both in vivo and vitro. Activation of Stat3 (p-Stat3) was induced by Shp2 depletion. Expression of p-Stat3 was negatively correlated with Shp2 expression in ESCC tissues. Furthermore, knockdown of Shp2 attenuated cisplatin-sensitivity of ESCC cells. Shp2 might suppress the proliferation of ESCC by dephosphorylation of p-Stat3 and represents a novel research field for targeted therapy.

## 1. Introduction

Esophageal cancer is the eighth most common cancer, and ranks as the sixth leading cause of cancer-related death worldwide [1]. Esophageal squamous cell carcinoma (ESCC) is the predominant histological subtype of esophageal cancer, representing up to 90% of all esophageal cancer cases in the “esophageal cancer belt” [2]. Despite the progression in diagnosis and multidisciplinary treatment, the overall survival of advanced ESCC has not significantly improved [3]. As the lack of knowledge about molecular alterations involved in the pathogenesis of ESCC, a thorough understanding of molecular mechanism on carcinogenesis of ESCC is vital for effective diagnosis and therapeutics of ESCC.

Protein tyrosine phosphatase Shp2 (Src-homology 2 domain-containing phosphatase 2), encoded by PTPN11, is a member of a subfamily of non-receptor tyrosine phosphatases (PTPs) that contains two Src-homology 2 (SH2) domains [4,5]. Human Shp2 was first cloned in the early 1990s [6]. In contrast to most of other phosphatases which play a negative role in proliferation pathways, Shp2 is critical for activation of several pathways, like mitogen-activated protein kinase (MAPK) and extracellular signal related kinase (Erk) [7,8]. Shp2 plays an important role in cell proliferation, cell differentiation, cell migration, and cell survival mediated by growth factors, cytokines signaling, and extracellular-matrix receptors [9,10].

Intriguingly, activation of Shp2 induced by PTPN11 mutations leads to development of Noonan syndrome and juvenile leukemias [11,12,13]. In addition, hyperactivation of Shp2 has been confirmed to be involved in the pathogenesis of several solid tumors, identifying that Shp2 acts as an oncogene. Mutant EGFR activated Shp2 is essential for mutant EGFR driven lung adenocarcinoma [14]. Shp2 plays an important role in tumor growth and invasion of glioblastoma via interaction with PDGFRα and Dyn2 [15]. In breast cancer, Shp2 participates in the maintenance of tumor-initiating cells and tumor growth by activation of stemness-associated transcription factors and MAPK [16,17]. In prostate cancer, Shp2 contributes to metastasis by enhancement of epithelial-to-mesenchymal transition [18]. Moreover, Shp2 promotes tumor growth and metastasis of liver cancer by activating Ras/Raf/Erk and Pi3K/Akt/mTOR signaling [19]. On the contrary, Bard-Chapeau et al. found that hepatocyte-specific Shp2 knockout led to the development of hepatocellular cancer (HCC) in mice via activation of Stat3 (signal transducer and activator of transcription 3), implying a tumor-suppressing role of Shp2 [20]. Moreover, the tumor suppressor role of Shp2 has been demonstrated in glioblastoma mutiforme [21] and metachondromatosis [22,23,24]. These studies reveal a dual role for Shp2 in tumorigenesis.

So far, the role of Shp2 in ESCC has not been investigated. In this work, we found that Shp2 was downregulated in human ESCC tissues. Loss of Shp2 promoted proliferation and chemoresistance of the ESCC cell line, suggesting that Shp2 might function as a tumor suppressor in ESCC.

## 2. Results

### 2.1. Shp2 Expression Is Frequently Reduced in Human ESCC Tissues and Predicts Better Prognosis of ESCC Patients

**Figure 1:**
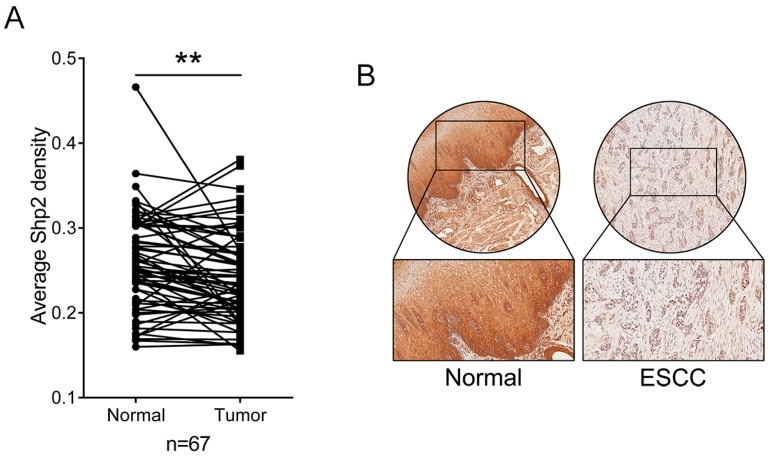

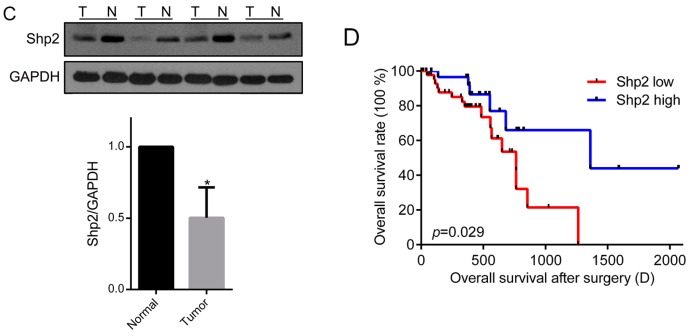
Expression of Src-homology 2 domain-containing phosphatase 2 (Shp2) is reduced in human ESCCs. (**A**) Comparison of Shp2 levels in 67 esophageal squamous cell cancer (ESCC) tissues and adjacent normal tissues using Immunohistochemical (IHC) staining and scoring; (**B**) Representative image of IHC staining of ESCC tissue and adjacent normal tissue; (**C**) Western blot assay of Shp2 expression in ESCC tissues; (**D**) Overall survival was compared between Shp2-high and Shp2-low groups by Kaplan-Meier survival analysis in clinical TCGA ESCC samples. (* *p* < 0.05, ** *p* < 0.01).

The expression levels of Shp2 were detected by immunohistochemistry (IHC) in ESCC tissues and adjacent normal tissues. Shp2 expression was remarkably decreased in ESCCs compared to adjacent normal esophageal tissue (0.2413 ± 0.0520 versus 0.2594 ± 0.0560, *p* = 0.003; Figure 1A,B and Table S1). Consistently, immunoblot analysis also demonstrated downregulated Shp2 expression in ESCC tissues relative to adjacent normal tissue (0.50 ± 0.21-folds, *p* = 0.019; Figure 1C). Moreover, Kaplan-Meier analysis of The Cancer Genome Atlas (TCGA) survival data for ESCC patients demonstrated that patients with low expression of Shp2 exhibited worse overall survival (OS) compared with Shp2-high patients (median OS 763 versus 1361 days, *p* = 0.029; Figure 1D). This suggested that the diminished expression of Shp2 was correlated with the development of ESCC.

### 2.2. Shp2 Knockdown Promotes ESCC Cell Proliferation In Vitro and In Vivo

**Figure 2:**
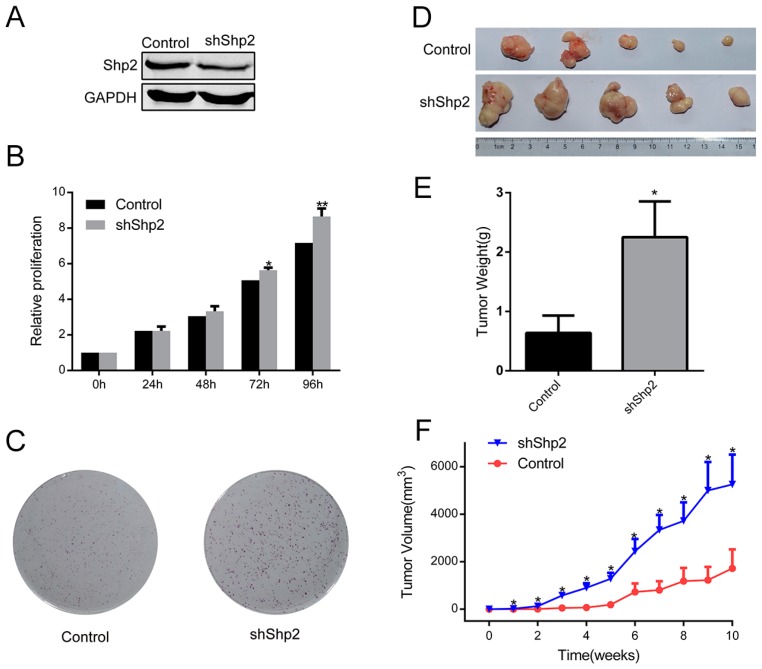
Shp2 knockdown enhances the proliferation of Eca109 cells in vitro and in vivo. (**A**) Eca109 cells were transfected with Shp2 shRNA or the matching control shRNA. The expression of Shp2 protein was analyzed by western blotting; (**B**) Proliferation of Eca109 shShp2 and control cells was evaluated by determining the cell viability by CCK8 Assay; (**C**) Representative images of cell colonies in control and shShp2 Eca109 cells; (**D**) Tumors derived from control versus shShp2 Eca109 cells at 10 weeks post inoculation are shown; (**E**) The weight of the tumor at 10 weeks was evaluated; (**F**) Tumor growth curves over 10 weeks are shown. (* *p* < 0.05, ** *p* < 0.01).

To illustrate the effect of Shp2 downregulation on ESCC cell behavior, a Shp2-specific shRNA lentivirus was utilized on Eca109 to generate Shp2-knockdown stable transfectants (Figure 2A). Shp2 depletion significantly enhanced proliferation of ESCC cell line Eca109 (Figure 2B). Moreover, Shp2-deficient ESCC cells displayed promoted ability of colony formation, producing more and bigger colonies (Figure 2C). We further evaluated the effects of Shp2-konckdown in vivo. The subcutaneous xenografts of Shp2-knockdown ESCC cells exhibited an elevated growth tendency and a larger tumor size than the control ESCC cells (0.64 ± 0.65 g versus 2.25 ± 1.34 g, *p* = 0.042; Figure 2D–F), indicating the suppressive effect of Shp2 on proliferation of ESCC cells in vivo.

### 2.3. Shp2 Negatively Regulates Activation of Stat3

It was reported that Shp2 expression resulted in negative regulation of Stat3 [25,26,27,28,29], which indicated that Shp2 expression was involved in proliferation of ESCC cells [30,31]. Additionally, Stat3 inhibition was responsible for tumor-suppressing role of Shp2 [20,21]. Therefore, we investigated whether Stat3 activation was affected by Shp2 expression.

**Figure 3:**
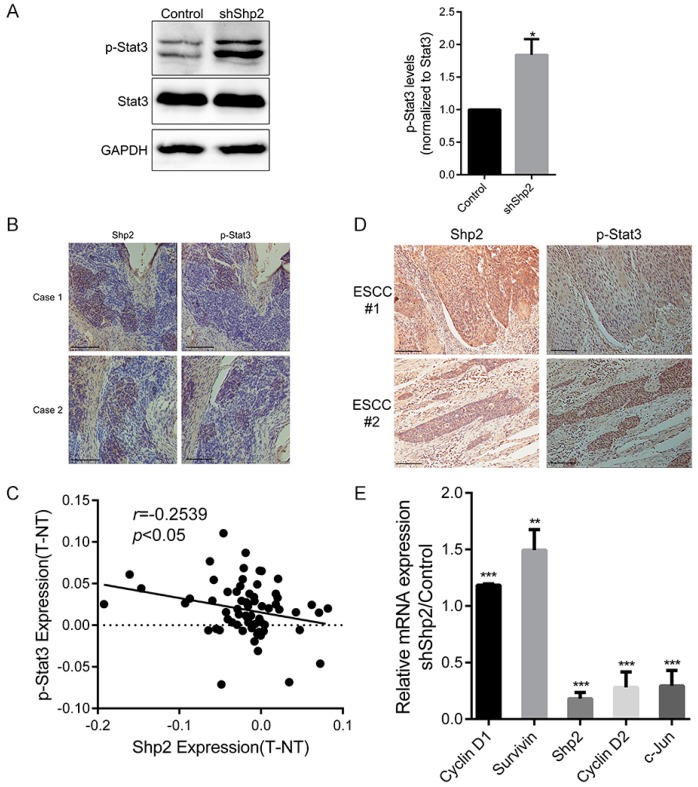
Shp2 knockdown promotes activation of Stat3 (signal transducer and activator of transcription 3) signaling. (**A**) p-Stat3 levels in shShp2 and control Eca109 cells were assessed by immunoblotting; (**B**) Stat3 phosphorylation negatively correlated with Shp2 expression in Xenograft tissues. Scale bar = 200 μm; (**C**) Inverse correlations were found between p-Stat3 level and Shp2 expression in ESCC tissues by IHC staining; (**D**) Representative image of IHC staining of ESCC tissue. Scale bar = 200 μm; (**E**) Relative mRNA expression of Cyclin D1, Survivin, Cyclin D2, c-Jun, and Shp2 in shShp2 or control cells was analyzed by real-time PCR. (* *p* < 0.05, ** *p* < 0.01, *** *p* < 0.001).

As expected, the interference of Shp2 remarkably upregulated p-Stat3 signal in ESCC cells, while Stat3 expression remained unaltered (1.84 ± 0.24-folds, *p* = 0.027; Figure 3A). Serial section of xenografts confirmed that suppression of p-Stat3 levels was abolished by Shp2 depletion (Figure 3B). IHC analysis of ESCC species showed an inverse correlation between Shp2 and p-Stat3 levels (*r* = −0.2539, *p* = 0.038; Figure 3C,D). Thus, Shp2 functions as a negative regulator for Stat3 activation in ESCC.

To identify effector molecules responsible for proliferation increments, we measured potential target genes involved in cell proliferation. Cyclin D1 and Survivin, downstream molecules of Stat3, were upregulated (1.18 ± 0.01-folds, *p* < 0.001; 1.49 ± 0.18-folds, *p* < 0.01) upon Shp2 depletion in ESCC cells, while Cyclin D2 and c-Jun expression were attenuated (0.28 ± 0.14-fold, *p* < 0.001; 0.29 ± 0.14-fold, *p* < 0.001; Figure 3E). We supposed that Shp2 regulated Cyclin D1 and Survivin expression through Stat3. Apparently, p-Stat3-mediated Cyclin D1 and Survivin expressions contribute to the promotion of cell proliferation induced by Shp2 depletion.

### 2.4. Shp2 Depletion Attenuated Cisplatin Sensitivity of ESCC Cells

**Figure 4:**
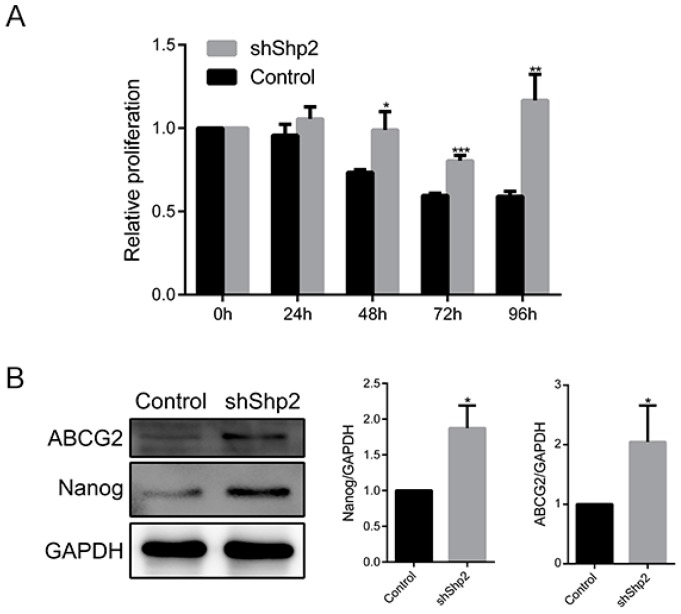
Knockdown of Shp2 reduced sensitivity of Eca109 cells to cisplatin treatment. (**A**) Proliferation of Eca109 shShp2 and control cells treated with cisplatin (10 µg/mL); (**B**) Expression of ABCG2 and Nanog in Eca109 shShp2 and control cells. (* *p* < 0.05, ** *p* < 0.01, *** *p* < 0.001).

To explore the potential function of Shp2 in sensitivity of ESCC cell to cisplatin, Shp2 knockdown ESCC cells were treated with cisplatin. ESCC cells with Shp2 knockdown displayed enhanced proliferation under cisplatin exposure compared with control cells (Figure 4A). As described previously, Shp2 depletion led to upregulation of Survivin (Figure 3E), which might contribute to the induction of cisplatin-resistance. Moreover, Shp2 depletion led to overexpression of ABCG2 and Nanog, responsible for drug-resistance phenotype in ESCC cells (2.05 ± 0.61-folds, *p* = 0.042; 1.87 ± 0.31-folds, *p* = 0.041, Figure 4B). Thus, Shp2 expression enhanced cisplatin sensitivity of ESCC cells.

## 3. Discussion

The role of Shp2 in tumorigenesis is controversial. Shp2, which is aberrantly activated in leukemia cells, was first recognized as a proto-oncogene in leukemogenesis [12,13]. Subsequent results highlight the oncogenic role of Shp2 in solid tumors. Shp2 inhibition suppresses EGFR mutant-induced lung adenocarcinoma by attenuating ERK1/2 and Src activation [14]. Dyn2 functions as a downstream modulator for Shp2 induced glioblastoma growth and invasion [15]. In breast cancer cells, Shp2 expression promotes tumor growth and progression through the Erk pathway [16,17]. Metastasis of prostate cancer is promoted by Shp2 via attenuation of PAR3/PAR6/aPKC polarity protein complex [18]. Shp2 overexpression promotes liver cancer growth and progression by activating Ras/Erk and PI3K/AKT [19]. By interaction with c-Src, Shp2 enhances migration and invasion of triple-negative breast cancer [32]. Ptpn11 gain-of-function mutation D61G induces chromosomal instability, resulting in tumorigenesis of leukemia, lymphoma, Lung adenomas, and skin papillomas in mice [33].

Nevertheless, recent studies suggest the tumor-suppressing role of Shp2. Deletion of Shp2 in hepatocytes leads to the development of spontaneous hepatocellular tumor and increased incidence of chemical carcinogen-induced HCCs, following hepatic inflammation and necrosis mediated by enhanced IL-6/Stat3 signaling [20]. In glioblastoma mutiforme (GBM) cells, Shp2 promotes EGFR and c-Met co-inhibition induced GBM cell death by inhibition of Stat3 activity [21]. Loss-of-function mutations in PTPN11 results in development of metachondromatosis (MC), while Shp2 represses the proliferation of progenitor cell population in cartilage [22,23,24]. Instead of Stat3 inhibition, Shp2 suppresses MC development via Erk-dependent inhibition of Indian Hedgehog (Ihh) production [22,24]. In the current study, we found that Shp2 expression was significantly reduced in ESCC tissues and associated with better prognosis, suggesting a tumor suppressor function of Shp2 in ESCCs. Consistently, we demonstrated that interference of Shp2 enhanced proliferation of ESCC cells in vitro and promoted the growth of ESCC xenografts in vivo.

Consistently, recent evidence has revealed the negative role of Shp2 for Stat3 activity in other tumors. Shp2 cooperates with c-SRC inmodulation of Stat3 phosphorylation in melanoma cells [34]. Stat3 was aberrantly suppressed in peripheral blood cells from Noonan syndrome patients with gain-of-function Shp2 mutations, while Stat3 activation was significantly attenuated by activated Shp2 in bone marrow cells [35]. In addition, Shp2 leads to Stat3 dephosphorylation of murine keratinocytes after ultraviolet B (UVB) irradiation, implying a tumor suppressor role for Shp2 in UVB-mediated skin carcinogenesis [36].

It has been demonstrated that Shp2 serves as a negative regulator for Stat3 signaling [25,26,27,28,29]. The inhibitory function of Shp2 on Stat3 activity requires Tyr-759 in the cytoplasmatic domain of gp130, an up-stream signal-transduction component of Stat3 [37,38]. Shp2 recruitments to gp130 moderate Jak activity and consequently lead to the attenuation of Stat3 signaling [39]. In addition, direct Stat3 dephosphorylation by Shp2 might be another conceivable mechanism causing decreasing Stat3 activation [35].

Stat3 activation is involved in proliferation of gastric cancer [40], colon cancer [41], glioblastoma [42,43], ovarian cancer [44], and breast cancer [43]. ESCCs cell proliferation was significantly inhibited by Stat3 knockdown with prolonged cell-doubling time compared to control cells [30]. Knockdown of Stat3 reduced ESCCs cell proliferation in vitro and in vivo [31], implying the positive role of Stat3 in ESCC growth controlling. Herein, we discovered an elevated p-Stat3 level after Shp2 depletion in ESCC cells. In addition, p-Stat3 levels were inversely correlated with Shp2 expression in ESCC tissues. Cyclin D1 and Survivin, target genes of p-Stat3 [45], were upregulated after Shp2 depletion, indicating the potential functional mechanisms mediated by p-Stat3 in ESCC. However, Cyclin D2 and c-Jun expressions were decreased after Shp2 knockdown. In the differentiation of embryonic stem (ES) cells, Shp2 regulates activity of Erk and Stat3 in a bi-directional manner [46]. We suspect that Shp2 depletion in ESCC might decrease p-Erk levels and lead to the downregulation of Cyclin D2 and c-Jun.

Shp2 activation contributes to drug resistance of several cancers. Shp2 facilitates IFN-γ resistance in hyperproliferating gastric cancer [47]. Shp2 is also required for proliferation in EGFR-inhibitor resistant non-small cell lung carcinoma (NSCLC) cells [48]. In hepatoma cells, Shp2 depletion leads to reduced proliferation and anchor-independent growth under sorafenib exposure [19]. Shp2 activation is essential to Growth-Factor-driven drug resistance [49]. Surprisingly, the current study revealed that ESCC cells with Shp2 knockdown showed elevated resistance to cisplatin, with upregulation of ABCG2 and Nanog.

Although the downregulation of Shp2 is demonstrated, the mechanism by which Shp2 expression is regulated remains unknown in ESCC. Shp2 activation mutation was detected in several leukemia and solid tumors [50]. Instead, Shp2 mutants in LEOPARD syndrome patients were catalytically defective and act as dominant negative mutations [51]. We analyzed mutation status of PTPN11/Shp2 in ESCCs using the cBioPortal for Cancer Genomics [52]. Only three missense mutations of PTPN11/Shp2 were detected in 225 ESCC patients. Maeshima et al. identified a putative enhancer in PTPN11 intron 1 which contained a glucocorticoid receptor-binding motif in rheumatoid arthritis fibroblast-like synoviocytes [53]. The glucocorticoid responsiveness of PTPN11 required differentially methylated CpGs for full enhancer function. Liu et al. found that alcohol treatment resulted in decreased methylation function of PTPN11/Shp2 in mouse embryos [54]. These data indicate that DNA methylation might participate in the regulation of PTPN11/Shp2 expression, which needs further research to be validated in ESCC.

Based on its interaction with oncogenic pathways, Shp2 is considered to be a potential target of cancer therapy. SHP099, selective and orally bioavailable Shp2 inhibitor, reduced Shp2 activity effectively through allosteric mechanism. Through suppression of Ras-Erk, SHP099 inhibits growth of multiple cancer cells in vitro and vivo [55]. Moreover, different Shp2 inhibitors are synthetized and exhibit tumor-suppression function on different cancers [56,57]. Due to the dual roles of Shp2 in tumorgenesis, more evidence is required to evaluate the security of Shp2-targeted therapy.

## 4. Materials and Methods

### 4.1. Patients and Samples

A total of 67 ESCC tissue samples were collected by surgery at Changzheng Hospital (Shanghai, China). No patients had received any other therapeutic intervention, such as chemotherapy or radiotherapy, prior to surgery. Peri-tumor normal tissues were diagnosed by three independent pathologists. The procedure of human sample collection was approved by the Ethics Committee of Changzheng Hospital (NO. 2016SL139, 3 June 2016).

### 4.2. Immunohistochemistry

The tissues were fixed in 10% neutral buffered formalin. Paraffin-embedding and 4 μm sections were performed following standard protocols. After deparaffinization, antigen retrieval was performed with sodium citrate buffer boiling for 5 min. Slides were blocked with 5% BSA at 37 °C for 10 min, incubated with diluted primary antibodies at 4 °C overnight, and then incubated with secondary antibody at 37 °C for 30 min. Antibody dilutions were 1:100 for Shp2 (cat no.sc280; Santa Cruz, Dallas, TX, USA) and 1:100 for p-Stat3 (cat no.4113 s; Cell Signaling, Danvers, CA, USA). The percentage of positively stained cells and staining intensity were assessed by Image-scope (Aperio Technologies, Inc., Vista, CA, USA). Integrated optical density of the positive-stained area was measured, and its ratio to total area of each photograph was calculated as average density. High expression of Shp2 is referred to as the average density is higher than the median value.

### 4.3. TCGA Cohort

An independent 76 patient specimens from The Cancer Genome Atlas (TCGA) database (https://tcgadata.nci.nih.gov/tcga) were applied to validate the prognostic value of PTPN11 in patients of ESCC. The statistical software X-tile (Yale University, New Haven, CT, USA) was used to determine the cutoff in the samples with Kaplan-Meier method (33 samples as the higher expression group and 43 samples as the lower) [58].

### 4.4. Cell Lines and Recombinant Viruses

ESCC cell line Eca109 was purchased from Cell Bank of Type Culture Collection of Chinese Academy of Sciences (Shanghai, China). Eca109 was cultured in high-glucose DMEM (Gibco, Grand Island, NY, USA) with 10% fetal bovine serum. Cells were cultured at 37 °C in a 5% CO_2_ humidified incubator. Lenti-virus expressing shRNA targeting Shp2 or control were generated using BLOCK-iT Lentiviral Pol II miR RNAi expression system kit (Invitrogen, Carlsbad, CA, USA; Table S2) and ViraPower Packaging Mix (Invitrogen). Lentivirus infection was utilized to establish Eca109 shshp2 and its control stable cell lines.

### 4.5. Cell Proliferation Assay

For cell proliferation analysis, shshp2-Eca109 and its control cells were seeded in 96-well plates at a density of 3 × 10^3^. Six hours later, medium was removed and replaced with fresh medium with or without cisplatin (10 µg/mL). ATP activity was measured using Cell Counting Kit-8 (Dojindo, Kumamoto, Japan) to assess their proliferation. Three independent experiments, each done in triplicate, were performed.

### 4.6. Colony-Formation Assay

Shshp2-Eca109 and control-Eca109 were harvested and seeded into 10 cm dishes (3000 cells). Cells were incubated at 37 °C in a 5% CO_2_ humidified incubator for 14 days. The cultures were fixed with 4% paraformaldehyde and stained with crystal violet.

### 4.7. RNA Extraction and Real-Time Quantitative PCR

Total RNA was extracted from cells using TRIzol Regent. Complementary DNA was synthesized from equivalent concentrations of total RNA using the Bestar qPCR RT Kit (DBI Bioscience, Shanghai, China) according to the manufacturer’s instructions. Real-time quantitative PCR was then performed using Bestar SybrGreen qPCR Mastermix (DBI Bioscience). Glyceraldehyde-3-phosphate dehydrogenase (GAPDH) was used as an internal control. The sequences of primers were shown in Table S3.

### 4.8. Western Blotting

Tissue samples and ESCC cells were homogenized and lysed in Radio Immunoprecipitation Assay buffer (RIPA) buffer with proteinase inhibitors. The lysates were resolved by way of SDS-PAGE (sodium dodecyl sulfate-polyacrylamide gel electrophoresis). Following electrophoresis, the proteins were transferred onto a nitrocellulose membrane. Primary antibodies were used in Western blotting according to protocols. Antibody dilutions were 1:10,000 for GAPDH (cat no.ab181602; Abcam, Cambridge, UK), 1:200 for Shp2 (cat no.sc280; Santa Crutz), 1:1500 for p-Stat3 (cat no.4113s; Cell Signaling), 1:1000 for Stat3 (cat no.12640s; Cell Signaling), 1:100 for ABCG2 (cat no.sc58222; Santa Crutz), and 1:2000 for Nanog (cat no.ab109250; Abcam).

### 4.9. Xenograft Transplantation

Xenograft transplantation was performed with five male nude mice aged six weeks. Cells stably expressing shRNA against Shp2 or luciferase (2 × 10^6^ cells in 200 μL PBS) were injected into the subcutaneous space of nude mice. Control-Eca109 and Shshp2-Eca109 were implanted into the right and left sides of the back, respectively. Tumor growth was evaluated by measuring the diameter of the tumor mass with calipers every week. Tumor formation was monitored over a 10-week period. Tumor volumes were calculated by the formula: V = diameter^3^ × 0.52. This study was approved by the Ethical Committee of the Second Military Medical University (NO. 20161002094, 17 June 2016).

### 4.10. Statistical Analysis

Statistical analysis in this study was performed with SPSS 18.0 (SPSS Inc., Chicago, IL, USA). Data were presented as “mean value ± SD”. The significance of mean values between two groups was analyzed by two-tailed Student *t*-test. Kaplan-Meier and log-rank analysis was used to evaluate the patient survival between subgroups. Pearson test was applied for analyzing the relationship ship between Shp2 and p-Stat3 levels. Statistical *p*-values < 0.05 were considered significant.

## 5. Conclusions

In the present study, we demonstrated that Shp2 was frequently downregulated in human ESCC tissues and associated with better prognosis, suggesting a tumor-inhibitory role of Shp2 in human ESCC. Knockdown of Shp2 promoted ESCC cell proliferation in vitro and the growth of xenografts in vivo, indicating the anti-proliferation role of Shp2 in human ESCC. In addition, cisplatin-resistance of Eca109 was significantly enhanced by Shp2 depletion. Mechanistically, we found that the knockdown of Shp2 led to overexpression of Cyclin D1 and Survivin. Moreover, ABCG2 and Nanog expressions were elevated, elucidating the mechanisms of cisplatin-resistance induced by Shp2 knockdown.

To our knowledge, this is the first research on the function of Shp2 in ESCC. Elucidating the tumor suppressor role of Shp2, as well as the underlying mechanisms, might lead to novel therapeutic strategies for the ESCC treatment.

## Supplementary Materials

Supplementary materials can be found at www.mdpi.com/1422-0067/18/1/134/s1.

## Abbreviations

ESCC	Esophageal squamous cell cancer
Shp2	Src-homology 2 domain-containing phosphatase 2
OS	Overall survival
Stat3	Signal transducer and activator of transcription 3
