# Supplementary material for: Shp2 Inhibits Proliferation of Esophageal Squamous Cell Cancer via Dephosphorylation of Stat3
Source: Int J Mol Sci. 2017 Jan 12;18(1):134. doi: 10.3390/ijms18010134 (PMC5297767; doi:10.3390/ijms18010134)
Supplement: Supplementary file 1 [file ijms-18-00134-s001.pdf]

Supplementary Materials: Shp2 Inhibits Proliferation of Esophageal Squamous Cell Cancer via Dephosphorylation of Stat3

Chen Qi, Tao Han, Hua Tang, Kenan Huang, Jie Min, Jing Li, Xinyu Ding and Zhifei Xu

Table S1. Clinical-pathological characteristics and Shp2 expression.

Variable	Shp2 Expression		*p*
	**Shp2-High Expression (*n* = 33)**	**Shp2-Low Expression (*n* = 34)**	
Median Age (range. years)	60.94 (43–80)	59.47 (41–81)	0.478
Gender			
Male	25	24	0.633
Female	8	10	
Grade			
G1	7	8	
G2	16	22	0.004
G3	10	4	
AJCC Stage			
I + II	13	20	0.112
III + IV	20	14	
T classification			
T1 + T2	6	13	0.069
T3 + T4	27	21	
N classification			
N0	14	18	0.389
N1	19	16	
Tumor size			
≥5.0 cm	18	18	0.895
<5.0 cm	15	16	

Shp2-high group (Shp2 expression in ESCC was higher than the median) and Shp2-Low group (Shp2 expression in ESCC was lower than the median) based on immunohistochemical analysis.

Table S2. shRNA sequences.

Name	Sequence (5′ to 3′)
Shp2-shRNA	GGGCCAGAGCAGTCAGTAA

Table S3. Sequence of primers for real-time PCR.

Primer	Sequence (5′ to 3′)
GAPDH forward primer	AGGTCGGTGTGAACGGATTTG
GAPDH reverse primer	TGTAGACCATGTAGTTGAGGTCA
Shp2 forward primer	CTGCCTCCACACCAGTGATA
Shp2 reverse primer	GGAGCCTGAGCAAGGAGC
Cyclin D1 forward primer	AGCTCCTGTGCTGCGAAGTGGAAAC
Cyclin D1 reverse primer	AGTGTTCAATGAAATCGTGCGGGGT
Survivin forward primer	GGCCCAGTGTTTCTTCTGCTT
Survivin reverse primer	GCAACCGGACGAATGCTTT
Cyclin D2 forward primer	GGACATCCAACCCTACATGC
Cyclin D2 reverse primer	CGCACTTCTGTTCCTCACAG
c-Jun forward primer	GGCGATTCTCTCCAGCTTCC
c-Jun reverse primer	TCGACATGGAGTCCCAGGA
